# A randomized controlled trial of an mHealth intervention for gay and bisexual men’s mental, behavioral, and sexual health in a high-stigma, low-resource context: Project Comunică protocol

**DOI:** 10.21203/rs.3.rs-3008174/v1

**Published:** 2023-06-26

**Authors:** Corina Lelutiu-Weinberger, Mircea Filimon, Donald Hoover, Mihai Lixandru, Lucian Hanu, Bogdan Dogaru, Tudor Kovaks, Cristina Fierbinteanu, Florentina Ionescu, Monica Manu, Alexandra Maris, Elena Pana, Cristian Dorobantescu, Adrian Streinu-Cercel, John Pachankis

**Affiliations:** Columbia University; Columbia University; Rutgers University; The Romanian Association Against AIDS; The Romanian Association Against AIDS; MozaiQ; MozaiQ; The Romanian Association Against AIDS; Insight; Insight; Mariș Alexandra - Cabinet Individual de Psihologie; Obregia Hospital; Data Center Solutions; The National Institute of Infectious Diseases; Yale University

**Keywords:** gay and bisexual men (GBM), Eastern Europe, HIV prevention, heavy alcohol use, stigma, mental health, behavioral intervention

## Abstract

**Background:**

The World Health Organization (WHO) reported that 80% of new HIV diagnoses in 2014 in Europe occurred in Central and Eastern Europe (CEE). Romania has particularly high HIV incidence, AIDS prevalence, and AIDS-related deaths. HIV incidence today in Romania is largely attributed to sexual contact among gay and bisexual men (GBM). However, homophobic stigma in Romania keeps GBM out of reach of the scant available prevention services and serves as a risk factor for HIV. The Comunică intervention delivers motivational interviewing and cognitive-behavioral therapy skills across eight live text-based counseling sessions. Preliminary evidence suggests that Comunică possesses promise for reducing GBM’s co-occurring mental (e.g., depression), behavioral (e.g., heavy alcohol use), and sexual (e.g., HIV-transmission-risk behavior) health risks in Romania and perhaps other similar high-stigma national contexts. This paper describes a randomized controlled trial (RCT) designed to test the efficacy of Comunică.

**Methods:**

To test Comunică’s efficacy, 305 GBM were randomized to receive Comunică or a content-matched education attention control condition. The control condition consists of eight time-matched educational modules that present information regarding GBM identity development, information about HIV transmission and prevention, the importance of HIV/STI testing and treatment, heavy alcohol use and its associations with HIV-transmission-risk behavior, sexual health communication, finding social support, and creating sexual health goals. Outcomes are measured pre-intervention (baseline), and at 4-, 8-, and 12-month follow-ups. The primary outcome is frequency of condomless anal sex acts with HIV-positive or unknown-status partners outside of the context of one’s own adherent PrEP use or primary partner’s adherent PrEP use or undetectable viral load in the past 30 days at each follow-up. Secondary outcomes include depression, anxiety, suicidal thoughts, heavy alcohol use, and HIV/STI testing; motivational and stigma-related mechanisms of intervention efficacy will also be examined.

**Discussion:**

If found to be efficacious, Comunică presents a scalable platform to provide mental, behavioral, and sexual health support to GBM living in Romania and similar high-stigma, low-resource areas within the CEE region and beyond.

**Trial registration:**

Registered April 11, 2019 to ClinicalTrials.gov Identifier: NCT03912753.

## BACKGROUND

The World Health Organization (WHO) reported that 80% of new HIV diagnoses in 2014 in Europe occurred in Central and Eastern Europe (CEE) [1]. Out of 15 surveilled European countries, Romania had the second-highest HIV incidence and AIDS prevalence, and the highest number of AIDS-related deaths between 2005–2014 [1]. After the first wave of the HIV epidemic in Romania, which occurred in children via nosocomial infection in the 1980s [2, 3, 4, 5, 6], a new wave, largely attributed to male-to-male sexual contact, emerged and is increasing [1, 7, 8]. The increased exposure of gay and bisexual men (GBM) to HIV and other STIs in the past two decades has purportedly resulted from more frequent travel to and from areas of high HIV prevalence outside of Romania, including many European Union (EU) states in Western Europe [9]; lack of sexual education in Romania in general [10, 11, 12] and specifically sexual education that responds to GBM’s distinct sexual health needs [13]; and pervasive stigma against GBM [14].

Despite these co-occurring threats resulting in increased HIV transmission risk, GBM remain under-prioritized in Romanian public health [2]. Furthermore, reporting of epidemiologic data might be unreliable in Romania due to ineffective surveillance systems and GBM’s anticipated stigma and normative identity concealment [15]. As a result, HIV transmission among GBM is frequently underreported and/or misclassified as heterosexual [1, 16, 17]. For instance, an independent European survey found that the number of HIV diagnoses self-reported by Romanian GBM respondents was 2.7 times the official national notification rate [17], suggesting that a significant proportion of HIV infections occurring among the GBM community might be attributed to heterosexual transmission and therefore yielding inaccurate guidance for national HIV-related priorities.

Best-available evidence suggests that HIV prevalence among Romanian GBM increased from under 10% in 2009 to around 20% in 2014 [18, 19, 20]. An international bio-behavioral survey of GBM found that Romania had the highest rate of unrecognized HIV infections across all included European countries [19, 20, 21, 22]. Untreated STIs represent a primary risk factor for HIV transmission [23], yet GBM-sensitive screening for STIs is rare in Romania. For example, of 629 GBM who sought an STI test across one year in the capital city of Bucharest, only 6.4% received both an anal swab and penile examination (compared to 72.4% in Amsterdam) – the lowest rate in all 40 cities participating in a European survey of GBM (*n* = 174,209). Furthermore, fewer than one-third of Romanian GBM had been screened for STIs in the prior year and fewer than half knew where they could access STI testing [20, 24].

Insufficient sexual health knowledge specific to GBM in Romania has direct implications for HIV transmission. A 2017 survey of 50 European and contiguous countries found that, of 2,002 Romanian GBM respondents, only 0.4% had ever used pre-exposure prophylaxis (PrEP) and 1.1% had ever used post-exposure prophylaxis (PEP), while 62% were unaware of PrEP and only 56% were knowledgeable that “undetectable = untransmissible.” Thirteen percent of respondents reported having at least two steady sex partners in the prior year with whom they did not use condoms (the third highest among the 50 surveyed countries); 32% reported having condomless sex due to lack of access to condoms (the seventh highest among the 50 surveyed countries) [25]. Of those who had never tested for HIV (approximately 50% in the Romanian sample), half (51%) did not know where to test (the fifth highest among 50 countries) [25].

In terms of mental and behavioral health needs that can co-occur with HIV-transmission risk among GBM [26, 27], European surveys indicate that 12% of Romanian GBM reported severe anxiety and depression during the prior two weeks and 21% had thoughts of self-harm [25]; 14% met criteria for alcohol dependency. Homophobic stigma is also a risk factor for HIV-transmission and co-occurring mental and behavioral health risks, as it keeps the majority of Romanian GBM hidden and out of reach of official HIV/STI surveillance and the few available prevention services. Depression, anxiety, heavy alcohol use, and homophobic stigma combine to create a syndemic affecting GBM [28, 29, 30].

Despite gains in some rights for LGBTQ individuals (e.g., decriminalization of homosexuality) in the past two decades, Romania remains one of the most structurally homophobic countries in Europe [31, 32], with structural homophobia being associated with low life satisfaction, poor mental health, social isolation, internalized homonegativity, and high degrees of identity concealment among GBM [15, 31, 33]. Identifying as an ethnic minority (e.g., Roma) may further amplify the odds of encountering hate speech and harassment [34] and intersectional challenges might exist for ethnic minority GBM in Romania [35]. A recent report by the European Union Agency for Fundamental Rights found significant physical or sexual attacks against LGBTQ people in Romania (15%) [34]. Of 28 EU Member States, Romania was among the top three countries in terms of the prevalence of hate-motivated physical or sexual attacks against LGBTQ people [34]. Perhaps as a further manifestation of structural stigma toward GBM, no governmental funds are currently allocated for HIV/STI prevention among GBM in Romania, despite the clear and increasing need for such prevention as outlined above [2, 18, 22, 36].

Evidence-based HIV-prevention interventions for GBM are not widely available in Romania, and are rare in the CEE region, where most HIV-related interventions have been developed for people who inject drugs [37]. One systematic review in 2015 identified only 24 HIV-prevention interventions for GBM in Europe [38], none of which appeared to have been implemented in Romania. To address this gap and respond to the interlocking challenges facing GBM in Romania that contribute to their increasing HIV-transmission-risk behavior, our team sought to adapt a promising intervention created for GBM in the U.S. [39], and which was recently pilot tested in Romania to support unmet needs of GBM [40], called Comunică.

### Comunică Intervention Background and Pilot

Comunică is based on the Information-Motivation-Behavioral Skills (IMB) model of health behavior change [41], which postulates that individuals must possess the requisite information for enacting sexual health; motivation to address their HIV-transmission-risk behavior, alcohol use, and mental health; and behavioral skills necessary for reducing risk behaviors. The Comunică intervention is guided by motivational interviewing (MI) principles and techniques [42], to provide accurate information about HIV transmission, heavy alcohol use, and local GBM-affirmative health resources (e.g., HIV-testing sites) and build motivation to improve behavioral skills via cognitive behavioral skills training (CBST) [43]. MI is an evidence-based form of person-centered therapeutic communication that privileges client values and preferences for change to help individuals resolve ambivalence and move toward their valued goals [41, 44]. For individuals who attain a high degree of motivation for change [45], CBST is used to promote awareness of contextual triggers and unhealthy behavioral patterns, and teach coping skills to reduce personal risk [46]. Given the stigmatizing context of Romania, the CBST skills in Comunică are presented in a manner that acknowledges the barriers posed by social stigma to GBM’s health, while empowering GBM to circumvent these barriers by building self-efficacy, learning effective communication, and implementing planful problem-solving [47, 48, 49]. In this way, Comunică also draws upon minority stress theory recognizing that stigmatizing societal contexts represent the ultimate source of health disparities affecting GBM [50]. Minority stress content informs the context in which the above psychoeducational information is presented, such as through a focus on identity development in the context of stigma and the ways in which minority stress affects mental, behavioral, and sexual health.

The Comunică intervention is delivered across eight sessions by a trained counselor via synchronous (i.e., live) text-based chat on a mobile-optimized website that also contains features for weekly tracking of HIV-transmission-risk behavior, heavy drinking, and mood. The study platform allows GBM randomized to the Comunică intervention to track their weekly number of condomless sex acts, number of partners, number of heavy alcohol use days, and positive and negative mood [51]. Review of this information with counselors during sessions is intended to support motivation and create contextually-informed behavior change goals. Previous research shows that mobile tools are the primary means for Romanian GBM to form and navigate social and sexual networks, especially given normative identity concealment [31], offering an ideal intervention platform [40]. While session content is driven by participant-selected priorities, these are discussed within the general theoretical and counseling frameworks of the Comunică intervention.

The feasibility, acceptability, and preliminary efficacy of Comunică was established in an open-trial pilot study with 43 young GBM in Romania (*M* age = 23.2, SD = 3.6, range 17–29) who reported condomless anal sex acts (CAS) with a male partner and at least five days of heavy drinking in the past three months [40]. Specifically, GBM who received Comunică reported, from baseline to a 3-months follow-up, significantly reduced depression, anxiety, and heavy alcohol use (*p* < 0.01), and increased condom use self-efficacy (*p* < 0.01), HIV-related knowledge (*p* < 0.01), and HIV testing (*p* < 0.05) [52]. While reductions in CAS trended in the expected direction from baseline to post-intervention follow-up (M = 14.7 vs. M = 10.8), the analyses were not sufficiently powered to detect significant differences. The Comunică intervention was created through in-depth consultation with 22 Romanian GBM and six community stakeholders (e.g., GBM advocates and service providers) and was based on a chat-based motivational interviewing intervention established in the U.S. [39].

### Study Objectives

The current study aims to test the efficacy of Comunică in reducing HIV-transmission-risk behavior (i.e., number of condomless anal sex acts with HIV-positive or unknown-status partners outside of the context of one’s own adherent PrEP use or primary partner’s adherent PrEP use or undetectable viral load in the past 30 days) in a randomized controlled trial among 305 Romanian GBM. Secondary outcomes include depression, heavy alcohol use, and HIV/STI testing. The control condition consists of eight time-matched educational modules that present information regarding GBM identity development, HIV/STI prevention, heavy alcohol use and its associations with HIV-transmission-risk behavior, sexual health communication, and the importance of social support, created in consultation with Romanian GBM community members and advocates. The educational modules for the control condition are hosted on the same study website as the Comunică intervention, but without access to the counseling or behavioral and mood tracking features.

This study also tests two sets of mechanisms of intervention efficacy, including (1) motivational mechanisms derived from the information-motivation-behavioral skills model [41, 44, 53] (e.g., knowledge of sexual health and alcohol use effects, motivation to reduce HIV risk and heavy alcohol use, and self-efficacy for safer sex and reductions in alcohol use) and (2) minority stress mechanisms derived from minority stress theory (e.g., rejection sensitivity, stigma consciousness, identity concealment). [54] If shown to be efficacious in this trial, the Comunică intervention offers a unique mental, behavioral, and sexual health intervention capable of future implementation in Romania and other high-stigma, low-resource national contexts.

## Methods

### Design

**Figure 1:**
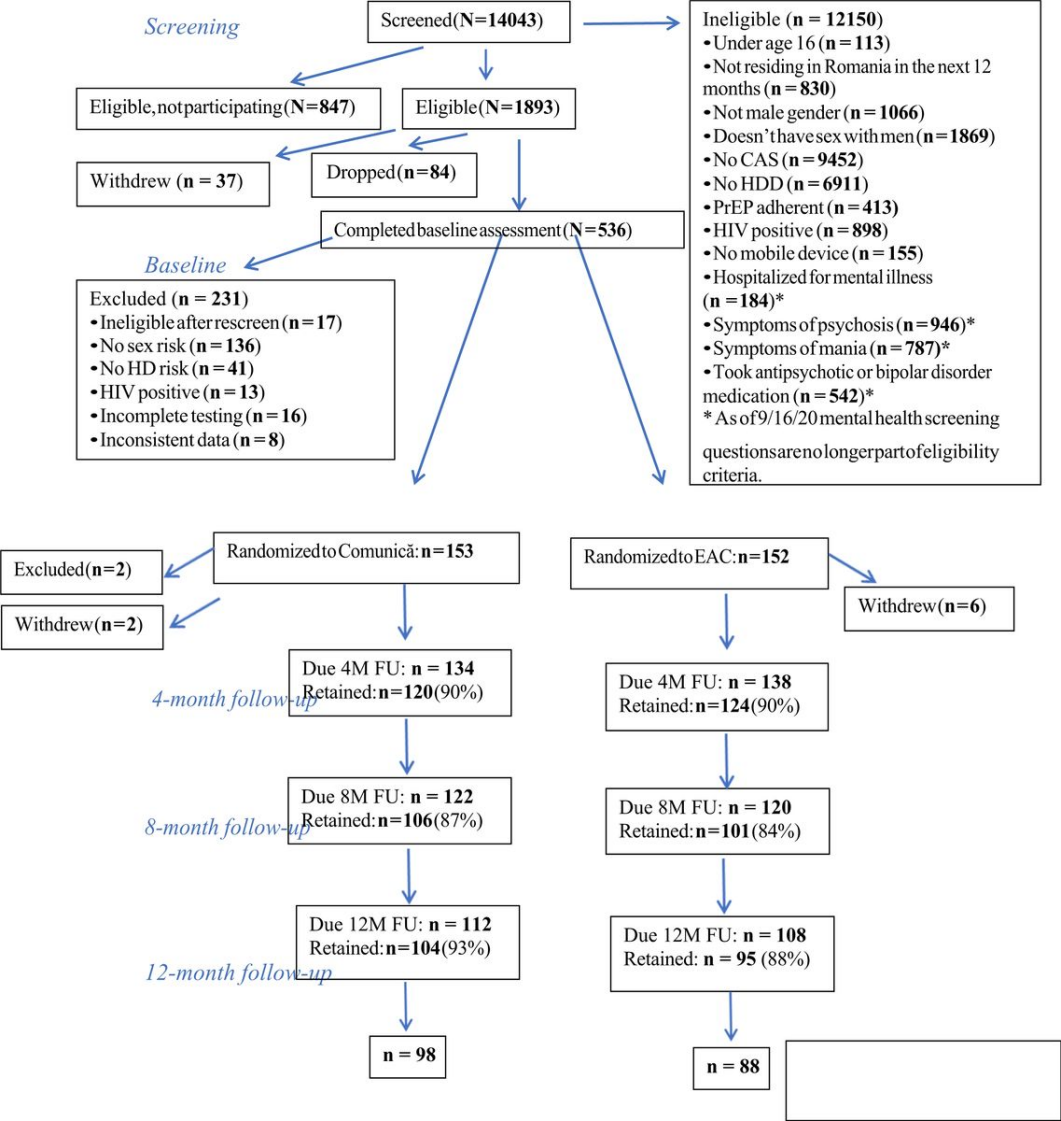
Consort diagram. M = months; FU = follow-up CAS = condomless anal sex HDD = heavy drinking days

In this randomized controlled trial, Comunică is compared to a content-matched education attention control (EAC) condition in changing (a) the primary outcome: frequency of condomless anal sex acts with HIV-positive or unknown-status partners outside of the context of one’s own or one’s primary partner’s adherent PrEP use or viral suppression and (b) secondary outcomes: depression, anxiety, suicidal thoughts, heavy alcohol use, and HIV/STI testing. Both the intervention and control groups received eight one-hour intervention sessions or educational modules, respectively, to be completed over the course of four months. Outcomes are measured at pre-intervention (baseline), and at 4-, 8-, and 12-month follow-ups. All participants self-administer at-home rapid testing for HIV and syphilis, and self-collect sampling (urethral, pharyngeal, rectal) for chlamydia and gonorrhea sent to a laboratory, at baseline and 12-month follow-up. Figure 1 outlines the flow of participants through the study.

### Study completion (assessment + testing)

#### Recruitment

Recruitment and enrollment for the study ended in January 2023. GBM living in, or within 40-miles from, 10 cities (i.e., București, Brașov, Timișoara, Cluj-Napoca, Iași, Constanța, Suceava, Craiova, Galați, Satu Mare) were recruited and screened. By selecting these cities, the study covers all regions of the country [55]. Collaborating LGBTQ advocacy organizations posted study ads on their websites, subscriber lists, and key virtual venues (e.g., Grindr, Facebook, Instagram). Study recruitment also relied on word-of-mouth; for instance, recruiters approached men in GBM-prevalent venues (bars; events; public cruising areas). Finally, enrolled participants were encouraged to share study information with peers. Still, 45% of participants were recruited on Grindr and 41% on Facebook.

#### Eligibility

##### Inclusion Criteria.

GBM were eligible if they reported: 1) male sex at birth and current male identity; 2) age ≥ 16; 3) ≥ 1 act of condomless anal sex acts with an HIV-positive or status-unknown male partner in the prior 30 days; 4) ≥ 1 heavy drinking days in the prior 30 days, (i.e., ≥ 5 standard alcoholic drinks on one occasion per month [56, 57]); 5) owning a mobile device (smartphone, tablet, laptop); 6) residence in Romania for the duration of study participation (12 months); 7) non-adherence to pre-exposure prophylaxis (PrEP); and were confirmed 8) to be HIV-negative upon testing at baseline.

##### Exclusion Criteria.

Participants were excluded if they demonstrated severe mental illness (e.g., active suicidality, psychosis, or mania).

#### Screening

Potential participants completed an eligibility screener on Qualtrics.com, a research-designated secure HIPAA-compliant online software [58]. Eligible GBM had the option to provide contact information at the end of the screener, in a separate survey, in order for research staff to later contact them to review the study in more detail and assess consent.

#### Consent

A research staff member contacted eligible participants by phone to review the consent form and verify age. Points of confusion were clarified and individuals still interested in participating submitted an electronic consent via Qualtrics [58]. The research staff outlined the steps to take place immediately following consent, namely that participants would receive a link to the baseline assessment, a numerical study identification number to be used for the duration of the study for confidentiality purposes, HIV/STI testing, and the study condition to which they were randomized. Participants were able to use any mobile device (smartphone, tablet, laptop) to complete their sessions.

#### Randomization

After baseline, participants were randomized in blocks of four and six to the Comunică or EAC condition, based on a list of random numbers generated by the study biostatistician. Stratification was made on baseline number of HIV-transmission-risk behavior sex acts (≤ 2 vs. > 2) and heavy alcohol use days (≤ 5 vs. > 5) in the past month. Counselors contacted participants assigned to the Comunică condition to schedule the first session, at which point they both logged onto the study platform. Group assignment was not masked from study staff given that all assessments were self-administered.

### Education Attention Control (EAC) Condition

The education attention control (EAC) condition consisted of eight educational modules based on the team’s HIV-prevention education with GBM in the U.S. and Romania [15, 39, 40, 48, 59, 60]: 1) GBM identity, 2) “HIV 101” (e.g., transmission risks, prevention, treatment), 3) the importance of HIV/STI testing and treatment, 4) alcohol and the body, 5) the role of alcohol in HIV-transmission-risk behavior, 6) HIV-status disclosure and sexual health communication, 7) finding social supports and safety, and 8) creating and reaching sexual health goals. The content was finalized in collaboration with staff of Romanian LGBTQ and sexual health organizations. To maximize participant engagement and learning, each module contained a 5-item quiz (with correct answers subsequently provided) and interactive exercises and vignettes prompting participants to provide answers based on their understanding of the topics and their own experience.

To minimize contamination, counselors who administered Comunică did not interact with EAC participants, and Comunică and EAC materials were only accessible by unique login credentials known only to participants.

### Counselor Training and Fidelity Monitoring

In 2019, prior to trial commencement, five psychologists living in Romania completed a 2-day training in the intervention. Two of these psychologists delivered the Comunică intervention in the pilot study [40] and three were recruited from a pool of 54 mental health professionals whom the PIs had previously engaged in a separate study [61]. The training included didactic and experiential components; a review of MI, CBST, the IMB model, and minority stress theory; unique facets of delivering the intervention via text; and reviewing vignettes from the pilot study. The counselors practiced delivering each session on the intervention platform, taking turns being a mock participant and counselor, and receiving biweekly remote video supervision from a clinical supervisor. At the start of the trial, the team reviewed all session transcripts. Once intervention fidelity was attained, the team randomly selected 52% of subsequent sessions to verify fidelity to the intervention content and adherence to motivational interviewing principles and techniques. As in the pilot study, the counselors translated each session transcript into English for the clinical team’s review.

### Study Assessments

Participants provided assessment data at baseline and 4-, 8-, and 12-month follow-up appointments, self-administered via Qualtrics [58] and a web-based platform designed specifically for this study to capture past-30-day sexual and alcohol use behaviors in a self-administered calendar review. Participants also completed biological testing for HIV, syphilis, gonorrhea, and chlamydia at baseline and 12-month follow-up appointments. Lastly, all retained participants completed an exit survey after their 12-month follow-up that assessed acceptability of the intervention. Participants were compensated in Romanian *lei* equivalent to $20, $25, $30, and $40 per baseline, 4-month, 8-month, and 12-month assessments, respectively, and $10 per session, based on each completed portion of the study. Measures are described below.

### Demographics

At baseline, as reflected in Table 1, participants indicated their age, sexual orientation, sex assigned at birth, current gender identification, income, residence (rural vs. urban), ethnicity, and education. Participants also indicated the age at which they attained sexual orientation development milestones (e.g., age of awareness of attraction to men) [62].

### Primary Outcome

#### HIV-transmission-risk behavior

This study’s primary outcome is the frequency of condomless anal sex acts with HIV-positive or unknown-status partners outside of the context of one’s own adherent PrEP use or primary partner’s adherent PrEP use or undetectable viral load in the past 30 days. Partner’s adherent PrEP use was verified by the participant having witnessed their partner taking their medication daily. Partner’s viral suppression in the past 30 days was verified by the participant having seen current test results. To report past-30-day sexual behavior, participants completed a self-administered online Time-line Follow-back (TLFB) interview [63, 64]. For each sexual act on each day, participants reported partner type (e.g. primary, casual), partner gender, partner HIV status and known viral suppression (if applicable), type of sexual behavior (e.g., insertive anal), condom use, and self and/or partner PrEP use. TLFB collects retrospective day-level data and has been validated for electronic self-administration [63]. The TLFB has good test-retest reliability, convergent validity, and agreement with collateral reports for sexual behavior [65, 66] and alcohol use [67]. For this study, we developed a mobile-optimized online platform for the TLFB and iteratively improved it during a usability testing phase with local GBM community members.

### Secondary Outcomes

#### Heavy Alcohol Use

The TLFB also asks participants to report their past-30-day heavy alcohol use, including whether it took place before and/or during sex. Participants also complete the 3-item Alcohol Use Disorders Identification Test (AUDIT-C) [68] a standardized measure of alcohol-related problems.

#### Mental Health

Participants complete the Center for Epidemiologic Studies – Depression (CES-D) scale as a measure of depression symptoms [69]; the Beck Anxiety Inventory (BAI) as a measure of anxiety symptoms [70], and the Suicidal Ideation Attributes Scale (SIDAS) [71] as a measure of suicidal ideation.

### Potential Intervention Mediators

#### Information-Motivation-Behavioral Skills

As informed by the Information-Motivation-Behavioral Skills Model, knowledge acquisition and motivation to reduce HIV-risk-transmission behavior and heavy alcohol use will be assessed as potential mechanisms of intervention efficacy. Information is measured using the Sexual Health Knowledge Questionnaire [72] and the Alcohol Attitudes Questionnaire [73]; motivation to reduce CAS and alcohol use is measured via the University of Rhode Island Change Assessment Scale (URICA) [74], and the Stages of Readiness and Treatment Eagerness Scale (SOCRATES) [75] scales, respectively; and behavioral self-efficacy to reduce HIV-transmission-risk behavior and heavy alcohol use risk is measured using the Safer-Sex Efficacy Questionnaire [76] and Confidence in Reducing Alcohol Use Questionnaire [77], respectively.

#### Minority Stress Pathways

We measured potential mechanisms suggested by minority stress theory [78] including sexual orientation concealment using the concealment motivation subscale of the Lesbian, Gay, and Bisexual Identity Scale (LGBIS) [79]; rejection sensitivity using the using the acceptance concerns subscale of the Lesbian, Gay, and Bisexual Identity Scale (LGBIS) [79]; internalized stigma using the internalized homonegativity subscale of the Lesbian, Gay, and Bisexual Identity Scale (LGBIS) [79]; assertiveness using the Rathus Assertiveness Schedule [80]; and social support using the Multidimensional Scale of Perceived Social Support [81].

Using question wording from the European MSM Internet Survey (EMIS) [21], participants indicate the frequency of their HIV and/or STI testing. Participants are asked whether they provided a blood sample to test for any STIs; whether their penis and anus were examined and swabbed as part of any STI testing in the past 4 months; and whether and if so when (e.g., past 7 days, 4 weeks) they had been diagnosed with chlamydia, gonorrhea, genital warts, herpes, syphilis, Hepatitis B or C, or urethritis.

### HIV/STI Testing, Counseling, and Linkage to Services

Participants complete HIV/STI testing after providing consent and completing the baseline assessment and the 12-month follow-up assessment. Specifically, a testing counselor affiliated with the study and based at a Romanian non-governmental organization specialized in HIV/STI prevention and treatment for GBM and other marginalized populations contacts the participant to provide two options: 1) mail an HIV/STI test kit to their home or 2) have the participant pick up the test at their offices. Participants receive a self-testing kit containing a rapid HIV/syphilis test, and swabs and urine collection container for pharyngeal, rectal, and urethral testing of chlamydia and gonorrhea. The testing counselor guides participants through the testing steps [82, 83]. The HIV and syphilis test results are available within 20 minutes of testing. Participants are required to take a photograph of the results (marked with their study ID) and upload it to a study platform. The chlamydia and gonorrhea self-collected samples are mailed back to the organization, which mails them for analysis to a laboratory. Upon receiving results for chlamydia and gonorrhea testing (usually within one week after lab receipt), the testing counselor communicates the results to the participant. For positive or inconclusive test results, the testing counselor provides participants with the name and contact information of the study-affiliated infectious disease provider in their area for confirmatory testing and treatment, as appropriate, and offers to assist with this linkage. Finally, participants were asked to complete a satisfaction survey at their 12-month follow-up [84].

### Data Analyses

#### Primary and Secondary Outcomes

It is hypothesized that participants randomized to the Comunică intervention, compared to those randomized to the EAC, will report significantly greater decrease on the primary outcome (HIV-risk-transmission behavior defined as condomless anal sex acts with HIV-positive or unknown-status partners outside of the context of one’s own adherent PrEP use or primary partner’s adherent PrEP use or undetectable viral load in the past 30 days) and secondary outcomes (depressive and anxiety symptoms, suicidality, heavy alcohol use, and HIV/STI testing outside of study testing) outcomes at 4-, 8-, and 12-month follow-up visits.

#### Sample Size Justification

As we approached the end of study recruitment, three factors transpired for estimation of power: i) While 305 participants were finally enrolled, we had estimated that we would likely only be able to recruit 288 subjects by 31 December 2022 (our date of termination of recruitment) and we therefore re-estimated power based on this number; ii) The overall participation rate for attending all three post-intervention visits was 86% (with a high proportion of the remaining sample completing two post-interventions assessments); iii) We had normative distributions of the study outcomes that could be used in establishing a study design and metrics for powering the primary outcome. To that end, we re-estimated the study power as described below. This new estimate was approved by the Data Safety and Monitoring Board (DSMB) and our program officer at the National Institute of Mental Health.

In the original proposal, a repeated measures mixed linear model (or generalized estimation equation) using all four study visits (baseline, and 4-, 8-, and 12-month follow-ups) was planned. However, the distribution of our primary outcomes (HIV-transmission-risk behavior) based on the data we now had near the end of the study was problematic for fitting this type of model, which is based on a central limit theorem assumption that may not manifest. Namely, there were two issues to consider: 1) at each follow up visit, over 50% of participants manifest no HIV-transmission-risk behavior creating a large point mass at the lower limit of 0. 2) On the other end, the distribution of HIV-transmission-risk behavior acts was very skewed with numbers of acts greater than 50 leading to skewness > 3, which again contradicts normality-based methods.

Although generally accepted approaches for power estimates for this situation do not readily exist, we found one approach that will satisfy assumptions for standard normal methods and that is also amenable to conducting a power estimation. That approach is to take the average behavior over all three follow-up timepoints (4-, 8-, and 12-month assessments) as a single within-person outcome, rather than evaluation of the repeated measures at 4-, 8-, and 12-month follow-ups as separate within-person outcomes. For example, if a person reported 0, 1, and 2 high risk acts respectively at the 4-, 8-, and 12-month follow-up visits, these would be summed together (0 + 1 + 2) = 3 and averaged over the 3 visits (3/3 = 1) as a single outcome of one high risk sex act over the previous 30 days per follow-up period. The details on what happened when we examined this outcome in our (still unblinded to intervention) data follow in the next paragraphs.

When we took the average of all three follow-up timepoints of HIV-transmission-risk behavior acts (i.e., during the previous four months), only 24% of participants had no HIV-transmission-risk behavior acts over the entire 12-month time period (i.e., the point mass at 0), which given our projected sample size of 288 (or 246 evaluable assuming 14% loss of data as we have been observing) is low enough to treat this average as a continuous variable in a normal approximation. However, due to a few individual(s) reporting very high numbers of risk acts (i.e., > 50), this variable was still skewed (> 3) so we capped (i.e., Winsorized) the maximum number of average per-assessment HIV-transmission-risk behavior acts at 15 acts, the upper 97th percentile of HIV-transmission risk behavior. When this was done, the HIV-transmission-risk behavior outcome was close enough to normal (i.e., skewness = 1.5) for the central limit theorem to apply to the linear model presented below.

The power estimation approach assumes that a linear model will be fit Y = a + b X + c T + ε

Where

Y = Averaged (during prior 4 months) prior 30 day high-risk sex behavior over 12-month behavior

a = intercept

X = baseline prior 30-day HIV-transmission-risk behavior acts

T = 0 for control, 1 for intervention

ε is random error with mean 0 and constant variance

And a, b, c are unknown parameters that are estimated in the model fit

The null hypotheses c = 0 will be tested with an overall two-sided type 1 error of 0.05.

Importantly for power estimation, the correlation of baseline and the average 12-month HIV-transmission-risk behavior acts was 0.37, which means that after adjustment for the pre-intervention behavior, the standard deviation of the post-intervention behavior would be the square root of (1–0.37^2^) = 0.93 that of the unadjusted outcome. Based on this and with 288 subjects, 86% of whom participated in all 3 visits, as we have been observing will happen (or conservatively 123 in each treatment arm), there is 80% power to detect an effect size of 0.33, slightly greater than our originally estimated effect size of 0.25–0.27 and at the upper end of the range of effect sizes found for behavioral interventions addressing multiple health outcomes among sexual minority men [85]. We believe that 0.33 represents a plausible effect size to detect in this trial given the strong distinction between the two intervention conditions, with one involving an active therapist-guided intervention and the other consisting of self-guided psychoeducation only. The standard deviation of the (Winsorized) averaged outcome over 12 months per-assessment HIV-transmission-risk behavior acts was 3.46 acts. Multiplying this standard deviation by the effect size of 0.33 gives 1.14 HIV-transmission-risk behavior acts. This means that the study will have 80% power to detect an overall mean reduction of 1.14 HIV-transmission-risk behavior acts in the prior 30 days per assessment period in the intervention compared to the control condition.

It should be noted that our final analysis will most likely incorporate the partial information from men with only 1 and 2 post-intervention follow-up visits through imputations, or more exactly adjustment of the partial information for number of follow-up periods reported, If so, this would increase power, albeit by a very modest amount.

#### Data preparation

Skewed variables will be recoded for analytic symmetry using appropriate log, square root, or other non-linear transformations. Should we fail to be able to find a transformation that achieves sufficient linearized normality, then robust generalized estimation equations (i.e., with logit or log link) will be fitted to dichotomized or count outcomes. We will also examine variable distributions, which may suggest dichotomous and multinomial recoding relevant to our primary research questions and would increase the statistical power of our models. Dependent and independent variable values will be cross-plotted as a function of time-in-study and summarized using parametric and non-parametric modeling methods such as loess curves. In addition to detecting trends and temporal patterns, graphic representations of time series data will provide knowledge of within- and between-individual variability of measurements. Results will be used to construct more complex cross-group/-time analytic models (below).

#### Analytic Plan

The analysis of the primary and secondary outcomes will use intent-to-treat with participants analyzed according to their original treatment assignment. SAS 9.4, SPSS 26.0, Stata and/or R software will be used for all analyses.

#### Comparability of Treatment Groups

Differences in baseline demographic characteristics between the two treatment arms will be assessed using appropriate graphical and statistical methods including summary statistics and *p*-values from exact, rank, chi-square, *t*-tests and ANOVA. Of note, as analyses progress, we will control for variables related to the study outcome in the analyses. We will also investigate if the randomization scheme was compromised.

For the HIV-transmission-risk behavior primary outcome (number of condomless anal sex acts in the past 30 days with HIV-positive or unknown-status partners outside of the context of one’s own adherent PrEP use or primary partner’s adherent PrEP use or undetectable viral load) analyses, the statistical significance threshold for an intervention (vs. control) arm effect will be a two-sided *p* ≤ 0.05. The primary outcome will be evaluated between the two arms at 4-, 8-, and 12-month follow-ups combined in a repeated measures analysis that adjusts for baseline behavior. This will be analyzed as using negative binomial regression with baseline, and 4-, 8-, and 12-months clustered within the same person. Main effect terms for 4-, 8-, and 12-months post-baseline (each timepoint vs. baseline) will be included in the model. A single interaction term between the 4-, 8-, and 12-month measures with the intervention arm will be included in the model to test for pooled post-baseline treatment arm differences. The relative number of HIV-transmission-risk behavior acts with a 95% confidence interval about his term will quantify intervention effect. Generalized estimating equations (GEE) with person as the cluster will be used to account for within-person repeated measure collinearity. As a sensitivity analysis, this will be repeated including all baseline covariates that are statistically associated (*p* < 0.05 to enter and *p* ≥ 0.10 to leave in a stepwise selection) with HIV-transmission-risk behavior in negative binomial GEE models with person as the cluster and adjusting for time of visit (e.g., 4-, 8-, and 12-months each vs. baseline). If there are excess zeros at each post-baseline visit, we will consider using a zero-inflated negative binomial model instead. However, this approach will split the intervention effect parameter into two models and thus may dampen power to detect statistical significance for an intervention that affects both parts. In this setting, we thus will more likely use the sensitivity analysis approach described below.

As a further sensitivity analysis, averaged HIV-transmission-risk behavior over all three follow-up visits (or two post-baseline visits if one visit is missing) will be used as the outcome in an ANCOVA linear regression model that adjusts for baseline HIV-transmission-risk behavior as a predictor and includes treatment arm assignment as a covariate. For those who are missing one follow-up visit, indicator variables as to which visit is missing will be included. The mean difference in HIV-transmission-risk behavior acts with a 95% confidence interval about his term will quantify intervention effect. This will be repeated including all baseline covariates that are statistically associated (*p* < 0.05) with HIV-transmission-risk behavior in stepwise selection (*p* < 0.05 to enter and *p* ≥ 0.10 to leave) into the above model. Should the negative binomial model described above fail to converge, this will become the primary analysis. The statistical significance threshold for the new (vs. control) intervention arm effect will again be a two-sided *p* ≤ 0.05.

The secondary outcomes of interest (all of these assessed at baseline and at 4, 8, and 12-month follow-up visits) are depression and anxiety symptoms, suicidality, and heavy alcohol use. Depression, as measured by CES-D, will be examined as a continuous variable and as a binary variable using the cutoff of ≥ 16 (indicating clinical depression). Anxiety, as measured by BAI, will be examined as a continuous variable and using the cutoff of ≥ 16 (indicating potentially concerning levels of anxiety). Suicidality, as measured SIDAS, will be examined as a continuous variable, as a binary outcome using the cutoff of ≥ 21 (indicating high risk of suicidality), as well as any score above 0. Heavy alcohol use, as measured by AUDIT-C, will be examined as a continuous variable and as a binary outcome using the cutoff of ≥ 4. Percentage of heavy drinking days in the past 30 days prior to the visit, as measured by the TLFB, will be examined as a continuous variable. Due to multiple comparison issues, these will each be tested individually using a two-sided Type-1 error of 0.01 and quantified using 99% confidence intervals. Levels of these measures at 4-, 8-, and 12-month follow-ups will be compared (adjusting for the level at the baseline visit) between the Comunică intervention and EAC in repeated measures analyses as described below.

For continuous outcomes that are heavily skewed to the right and without an excessive point mass at 0 for 4-, 8-, and 12-month follow-ups (e.g., the BAI or number of heavy drinking days), a similar approach to that described for the primary outcome analyses will be used. For continuous outcomes that are not heavily skewed to the right at 4-, 8-, and 12-month follow-ups (e.g., CES-D or AUDIT-C) repeated measure linear regression mixed models will be fitted for outcomes at baseline and 4-, 8-, and 12-month follow-ups with subject intercept as a fixed effect, main effects for 4-, 8-, and 12-month follow-ups, and a single interaction term between treatment arm assignment and the timepoint being follow-up. In sensitivity analyses, this will be repeated including all baseline covariates that are statistically associated (*p* < 0.05) with the outcome in models using stepwise selection (*p* < 0.05 to enter *p* ≥ 0.10 to leave). The mean post-intervention difference in the outcome between the treatment arms with 99% confidence intervals about this term will quantify intervention effect. Binary outcomes (e.g., HIV-transmission-risk behavior > 0, CES-D ≥ 16, SIDAS ≥ 21, SIDAS ≥ 0, AUDIT-C ≥ 4) will be analyzed in repeated measures models using the baseline visit and follow-up visits at 4-, 8-, and 12-month follow-ups. Repeated measures generalized estimation equations will be fit about individual as the cluster using a logit link. The visit number (i.e., 4-, 8-, and 12 months vs. vs. baseline) and treatment arm assignment will be included as main effects, as will the baseline level of the outcome being modeled. In sensitivity analyses, this will be repeated including all baseline covariates that are statistically associated (p < 0.05) with the outcome in models using stepwise selection (p < 0.05 to enter p ≥ 0.10 to leave). The intervention effect will be quantified by odds ratios with 99% confidence intervals. Finally, we will also use exact tests to compare treatment arms for having been ever diagnosed during the single timepoint 12-month study follow up with HIV, syphilis, chlamydia, and gonorrhea.

#### Mediation analyses

In our mediation analyses, we will examine whether changes in the proposed mediators (e.g., self-efficacy for condom use or heavy alcohol use prevention, identity concealment, internalized homophobia) precede and statistically mediate intervention effects consistent with our IMB and minority stress models. We will use path analysis/structural equation modeling to model and assess the size of the indirect effect from intervention condition to 12-month outcomes through mediators assessed at 4- and 8-months controlling for baseline effects of these mediators.

### Ethical Research Conduct

#### Overall Assessment of Risk

Participants are at minimal risk for harm associated with study participation. Although unlikely, risks of this study are potential emotional discomfort from completing the assessments and/or the intervention sessions, and breaches of confidentiality. Additionally, participants may experience discomfort during the HIV/STI testing and/or emotional distress when receiving positive test results. All possible steps are taken to minimize such risks through our carefully designed protocols, and staff training and fidelity monitoring. All study staff are trained in and follow study clinical protocols to protect against risks. Risks are being monitored and addressed (as below) during assessments (via direct real-time triggers signaling emotional distress), counseling sessions and in-between assessment points (via communication from participants in both arms with study staff).

#### Risk of Emotional Discomfort

Participants may experience emotional distress associated with the study content. The consent document indicates that participants do not have to respond to any questions they do not wish to answer and may discontinue their participation at any time, while being compensated for the portions of the study they completed.

At each assessment point, participants complete the SIDAS, with a score of 21 or above triggering an email to the project staff, who immediately alerts the study counselors who contact the participant to assess their wellbeing and potential need for immediate intervention in case of severe distress. All counseling resources are available to participants regardless of ability to pay.

Should participants experience discomfort during assessments and/or sessions, they are contacted as above to assess their mental state, need for referral or immediate intervention, and capacity to continue in the study. Based on the counselors’ determination, the participant may continue with the study (if there is no concern for imminent harm or lack of capacity to consent) or be referred to local in-person or telehealth LGBTQ-affirmative counseling services. For any person judged to be a danger to self or others or be in imminent need of medical or mental health services, the project staff contacts local emergency services to intervene.

#### Risk of Physical Discomfort

Biological testing for HIV, syphilis, chlamydia, and gonorrhea may be associated with physical discomfort from the finger prick or incorrect swabbing, of which participants are informed during the consent process. Appropriate testing procedures and risk-reduction counseling are provided to all participants at time of testing at baseline and 12-month follow-up. Staff are available by phone and email to answer questions participants may have about performing biological testing. These tests are routinely done and, therefore, potential risks are no greater than those encountered during routine medical exams. At the start of the study, we trained 10 infectious disease physicians from the 10 study cities of București, Brașov, Timișoara, Cluj-Napoca, Iași, Constanța, Suceava, Craiova, Galați, and Satu Mare. These physicians agreed to provide confirmatory testing and treatment to participants who test positive for HIV or other STIs during the study.

#### Risk of Breach of Confidentiality

Prior to any assessments, all participants are assigned a study identification number (ID). The name-ID link is kept under electronic password and firewall protection in one of the PIs’ offices at the [blinded for review] research space. Electronically-signed consent forms are kept in a database separate from data, under password protection. Records are kept confidential and information provided by study participants is not released to outside sources unless written consent is provided by the study participant, or it is required by law (e.g., suspicion of child abuse, elder abuse, and threat of imminent action on suicidal or homicidal ideation) or to protect participant well-being (e.g. in the event that immediate local intervention is required following a safety assessment). The online intervention platform is only accessible to study staff and participants, whose log-in information is not linked to any identifying information. All participants are required, as part of the consent process, to upload to REDCap a photograph of their HIV and syphilis test paddle showing the results and label marked with their unique study ID [86]. Lastly, all procedures are being monitored by the Human Subjects Protection Programs at [blinded for review] and the study’s Data and Safety Monitoring Board.

#### Risk versus Benefit

Given the public health significance addressed by this first study of its kind in CEE, the social, psychological, and physical risks reviewed above are likely to be outweighed by the new knowledge gained regarding the efficacy of this highly scalable and portable approach to reducing GBM’s HIV-transmission-risk behavior and increasing wellbeing. Preventing the further spread of HIV presents clear public health implications, especially in high-stigma low-resource contexts such Romania and other CEE countries. By participating in this study, participants may gain insight into their sexual, behavioral, and mental health that could lead to sustained behavior change. Additionally, study participants may reduce their risk of acquiring HIV/STIs through involvement in our counseling/education sessions and behavioral risk tracking. Of note, participants are the first to be involved in at-home STI testing in Romania, a procedure they may adopt routinely in their lives beyond the study, both reducing their personal health risk and potentially promoting these protective practices within their networks.

#### Data Safety Monitoring Plan (DSMP)

Any unexpected or serious adverse events (e.g., hospitalization) that occur during the course of the study will be reported by the contact PI, [blinded for review], to the Committee on Human Research (IRB) at [blinded for review University] in accordance with current guidelines for reporting adverse events. She [blinded for review], the Co-Principal Investigator and clinical psychologist, meet bi-weekly to discuss study progress, and address participant safety immediately as issues arise (e.g., reported suicidal ideation).

#### Data and Safety Monitoring Board (DSMB)

A five-member monitoring committee has been convened to determine safe and effective conduct and recommend conclusion of the study if significant risks develop or if the trial is unlikely to be concluded successfully. On May 6, 2019, the team held the first Data Safety Monitoring Board meeting and has been convening annually since. The five members have reviewed and approved the study design and procedures and a plan for monitoring study data and interim outcomes. Starting in Year 2 and annually thereafter, the study statistician has prepared and presented to the DSMB both open (pooled) and closed (stratified and unidentified treatment arm) reports on the following data, by arm: suicidality, HIV/STI test results, depression, heavy alcohol use, and HIV-transmission-risk behavior. Upon review of study progress, the DSMB provides a determination about study continuation to the principal investigators, who share it with NIMH in their annual progress report.

## Discussion

This study is the first to test the efficacy of an intervention with potential to simultaneously support the sexual (e.g., HIV-transmission-risk behavior), behavioral (e.g., heavy alcohol use), and mental (e.g., depression) health of GBM in Central and Eastern Europe, using motivational interviewing support and sensitivity to the high-stigma context of the region [39, 40]. The resulting intervention holds promise for building a bridge from initial online counseling support to on-the-ground service utilization. If efficacious and cost-effective, the Comunică intervention presents a scalable platform to address HIV/STI risk and provide behavioral and mental health support to GBM in other high-stigma, low-resource areas in the region (e.g., Ukraine, Poland) and the U.S. (e.g., rural areas). This study will also generate intervention content and clearly defined protocols that can be easily replicated by international service providers who do not operate in a research capacity.

## Figures and Tables

**Table 1.** Participant demographics (*N* = 305).

	n (%)

**Age**	
16–29	232 (76)
30–39	56 (18.5)
40–49	16 (5.2)
50+	1 (.3)

**Sexual identity**	
Gay	205 (67)
Bisexual	92 (30)
Queer	2 (.5)
Pansexual	2 (.5)
Uncertain	4 (2)

**Education**	
High school or less	78 (26)
Vocational studies	9 (3)
Some college	108 (35)
College degree	57 (19)
Graduate degree	53 (17)

**Relationship status**	
Single and not dating	97 (31)
Dating	98 (32)
In a serious relationship	110 (37)

**Ethnicity**	
Romanian	279 (91.7)
Hungarian	22 (7)
Roma	3 (1)
Other	1 (.3)

**Employment**	
Full time	149 (49)
Part time	18 (6)
Unemployed	15 (5)
Student	121 (39)
On disability	2 (1)

**High school location**	
Small town	161 (53)
Medium to large town	144 (47)

**Age attracted to men**	
4–14	230 (75)
15–25	72 (24)
26–34	3 (1)

## Data Availability

Not Applicable

## Abbreviations

AUDIT-C: Alcohol Use Disorders Identification Test
BAI: the Beck Anxiety Inventory
CAS: Condomless anal sex acts
CBST: Cognitive behavioral skills training
CEE: Central and Eastern Europe
CES-D: Center for Epidemiologic Studies – Depression scale
DSMB: Data Safety and Monitoring Board
DSMP: Data Safety Monitoring Plan
EAC: Education attention control
EMIS: European MSM Internet Survey
EU: European Union
GBM: Gay and bisexual men
GEE: Generalized estimating equations
ID: Identification
IMB: Information-Motivation-Behavioral Skills
LGBIS: Lesbian, Gay, and Bisexual Identity Scale
MI: Motivational interviewing
PEP: Post-exposure prophylaxis
PrEP: pre-exposure prophylaxis
RCT: Randomized controlled trial
SIDAS: Suicidal Ideation Attributes Scale
SOCRATES: Stages of Readiness and Treatment Eagerness Scale
TLFB: Time-line Follow-back
URICA: University of Rhode Island Change Assessment Scale
WHO: World Health Organization
